# Time to act: A rubric-based approach for institutionalizing justice, equity, diversity, and inclusion

**DOI:** 10.1017/cts.2023.599

**Published:** 2023-07-24

**Authors:** Marie K. Norman, Thomas R. Radomski, Chelsea N. Proulx, Doris M. Rubio, Tasha L. Alston, Colleen A. Mayowski

**Affiliations:** 1 Institute for Clinical Research Education, University of Pittsburgh School of Medicine, Pittsburgh, PA, USA; 2 Division of General Internal Medicine, University of Pittsburgh School of Medicine, Pittsburgh, PA, USA; 3 Clinical and Translational Science Institute, University of Pittsburgh, Pittsburgh, PA, USA

**Keywords:** Rubric, diversity, equity, inclusion, justice, JEDI, evaluation

## Abstract

Attacks on minoritized communities and increasing awareness of the societal causes of health disparities have combined to highlight deep systemic inequities. In response, academic health centers have prioritized justice, equity, diversity, and inclusion (JEDI) in their strategic goals. To have a sustained impact, JEDI efforts cannot be siloed; rather, they must be woven into the fabric of our work and systematically assessed to promote meaningful outcomes and accountability. To this end, the University of Pittsburgh’s Institute for Clinical Research Education assembled a task force to create and apply a rubric to identify short and long-term JEDI goals, assess the current state of JEDI at our Institute, and make recommendations for immediate action. To ensure deep buy-in, we gathered input from diverse members of our academic community, who served on targeted subcommittees. We then applied a three-step process to ensure rapid forward progress. We emerged with concrete actions for priority focus and a plan for ongoing assessment of JEDI institutionalization. We believe our process and rubric offer a scalable and adaptable model for other institutions and departments to follow as we work together across academic medical institutions to put our justice, equity, diversity, and inclusion goals into meaningful action.

## Introduction

The high-profile murders of unarmed Black men and women by police, attacks on Asian Americans, Jewish Americans, and members of the LGBTQ + community, and the disturbing rise of White nationalism in the US and abroad have brought increased attention to persistent strands of bigotry and violence in our society. At the same time, striking racial and ethnic health disparities laid bare by the COVID-19 pandemic have prompted the health professions to take a hard look inwards, igniting a wave of health disparities and equity research [1,2]. As the moral imperative to combat discriminatory practices becomes more and more clear, the benefits of diversity and inclusion are also becoming increasingly obvious, with team science research highlighting the fact that diverse teams, working in inclusive and equitable environments, produce better science [3–5].

In recognition, the National Institutes of Health (NIH) and National Center for Advancing Translational Science (NCATS) have prioritized justice, equity, diversity, and inclusion (JEDI) in their strategic goals, and the Clinical and Translational Science Award (CTSA) consortium has followed suit [6–8]. In 2021, a task force formed by the CTSA in support of structural and transformational JEDI initiatives identified five guiding principles and four focus areas, with 86% of CTSA consortium members surveyed reporting a commitment to advancing JEDI [9].

Despite these promising signs, there is also widespread acknowledgement (including by the CTSA task force) that change related to advancing JEDI has been too slow [10]. JEDI initiatives are often instituted in a top-down manner without the full understanding or backing of faculty and staff, and sometimes without meaningful follow-through [11], making it difficult for such initiatives to gain traction. In many cases, faculty from minoritized groups are given primary responsibility for JEDI programs, adding to the diversity tax [12] they already pay and at times even negatively affecting their career progression [13]. Additionally, JEDI initiatives frequently take the form of stand-alone programs (trainings on implicit bias, for instance). While important and often innovative, these programs may not be sufficiently integrated into the day-to-day work of academic medicine to make a sustained difference [14,15]. Many JEDI programs lack operationalized outcomes[16] and thus struggle to show demonstrable progress. Moreover, what progress has been made in the JEDI space has recently come under direct threat, with a powerful political backlash in several states [17,18] intent on rolling back JEDI initiatives and altering school and university curricula to avoid uncomfortable reckoning with racism and other forms of systemic inequality and discrimination [19,20]. Finally, even where the institutional *will* to address JEDI is there, the *way* – i.e., the path forward – may not always be clear. Indeed, departments and programs may struggle to understand their own responsibilities within the larger JEDI mission.

For the CTSA commitment to JEDI to have tangible and meaningful effects, JEDI must be *institutionalized*: woven into everything we do in our schools and departments.

Institutionalization ensures that JEDI initiatives are substantive and makes DEI less vulnerable to political vicissitudes. Commitments to JEDI must also have the *buy-in* of faculty and staff as well as leadership. Forging meaningful culture change requires that all key groups, each with its own expertise and perspectives, are involved in the discussion of goals and priorities [21]. Moreover, the outcomes we desire must be operationalized and *assessed* to show results and promote accountability [21,22].

In this paper, we describe the rubric-based approach we took at the University of Pittsburgh’s Institute for Clinical Research Education (ICRE), the goal of which was to institutionalize JEDI, build bottom-up buy-in, enable assessment, and ensure accountability. We describe the process of forming a task force to develop our ICRE-JEDI rubric and the steps we took in adapting and applying it to measure our progress and set strategic priorities. We believe this process can be readily adopted by other CTSAs and institutions in academic medicine.

## Methods

The Institute for Clinical Research Education is a multidisciplinary institute that provides training in clinical and translational science and medical education. Our mission statement reads: “The ICRE, an avowed anti-racist organization, seeks to improve health outcomes, practice, and policy by creating an equitable, inclusive environment dedicated to educating the next generation of clinical and translational researchers and medical educators [23].” Committed to ensuring that the ICRE does not simply espouse but actually *lives* this mission statement, we assembled a task force in April 2022 charged with assessing the current state of JEDI at the ICRE and providing recommendations to increase JEDI institutionalization.

### Mission of the Task Force

The task force’s work focused on customizing a rubric designed to assist the ICRE in assessing its current state of JEDI institutionalization. The task force was given this charge:

- **Adapt** to the specific context of the ICRE a rubric developed to assess JEDI along six dimensions: Philosophy & Mission, Faculty Support, Curriculum, Staff Support, Student Support, and Administrative Leadership.
- **Apply** the rubric to determine the ICRE’s current level of JEDI development in each rubric dimension along a continuum of Emerging → Developing → Transforming.
- **Recommend** components that the ICRE should focus on to improve its institutionalization of JEDI.
- **Report** the findings to ICRE leadership and create a plan for implementation.

### Genesis of the Rubric

The ICRE’s customized rubric was informed by a rubric developed by the New England Resource Center for Higher Education (NERCHE). The NERCHE rubric was designed to help colleges and universities self-assess diversity, equity, and inclusion at the institutional level [24]. The NERCHE rubric identifies six dimensions for assessment: Philosophy & Mission, Faculty Support, Curriculum, Staff Support, Student Support, and Administrative Leadership. In developing our rubric, we retained these six dimensions and the three stages of development used by NERCHE to assess progress: Emerging, Developing, and Transforming. Before the rubric was ready for adaptation by the task force, Drs. Norman and Mayowski reviewed each dimension, keeping only those components relevant to the ICRE’s administrative context and education-focused work.

### Forming the Task Force

The Director of the ICRE (Rubio) and the chair of the task force (Mayowski) then assembled the task force members. In keeping with Fatima Cody Stanford’s advice in “The Importance of Diversity and Inclusion in the Healthcare Workforce” that partners from all levels of the organization and all key groups be included, Drs. Rubio and Mayowski selected ICRE members who:

- Represented the full spectrum of ICRE constituencies (faculty, staff, students, alumni, administration).
- Came from backgrounds underrepresented in academic medicine [25], such as underrepresented racial and ethnic groups, lower socioeconomic backgrounds and first-generation college educated, and fluid gender identities, as well as backgrounds well-represented in academic medicine.
- Had deep experience with the inner workings and daily activities of the ICRE.

Colleagues who accepted the invitation were each assigned to a committee that would concentrate on one of six rubric dimensions. Because we wanted task force members to speak freely without risk of offending Institute leadership, the director of the ICRE abstained from serving on the task force.

### Adapting and Approving the Rubric

Dr Mayowski organized and led two task force kickoff meetings (April 26 and May 2, 2022) to enable all members to attend. At this meeting, task force members were introduced to the rubric, assigned to a committee focused on one of the six dimensions, and given their charge. Each committee was given the freedom to meet at dates and times convenient for them but was encouraged to complete their work within a three-month period. We limited each committee to three members to facilitate the scheduling of meetings and speed of the deliberative process. Each task force member served on just one committee to prevent overburdening participants and to minimize the effects of the minority tax [26]. There were two exceptions to this rule: one member (to whom the minority tax did not apply) was asked to serve on two committees. The task force chair served on every committee, where she helped guide each committee through their deliberative process and served as a communication hub for sharing knowledge among committees. At committee meetings, members methodically and collaboratively reviewed the components of their assigned dimension and made further adaptations to fit the ICRE context, including removing components that did not apply, adding components that were not part of the original NERCHE rubric, and differentiating meaningfully distinct ICRE program elements (for instance, the committee working on the Curriculum dimension updated one component to specifically differentiate between course level attention to JEDI and master’s degree track level attention to JEDI). They also worked to operationalize each component so it could be assessed. For example, the committee in charge of the Faculty Support dimension operationalized the Faculty Knowledge and Awareness component at the Emerging stage to say, “Less than 60% of faculty members have completed formalized, evidence-based JEDI training or workshops sponsored by a credible organization or source.” (Appendix. ICRE-JEDI Rubric.) Although each committee operated independently of the others, they had access to the others’ work through a master document maintained by the task force chair on a shared drive. Additionally, the presence of the task force chair, who had visibility into all the committees, helped to ensure that each committee’’s rubric descriptions were consistent in length and tone.

### Applying the Rubric

When each committee was satisfied that their dimension was customized to the ICRE context, they applied the rubric by evaluating the current state of the ICRE along the three developmental stages as Emerging, Developing, or Transforming. A finding of Emerging meant that the ICRE either had insufficient information to draw a conclusion or was just beginning to recognize JEDI as a strategic priority and was gathering resources and building constituencies for this effort. A finding of Developing meant that the ICRE was focused on ensuring the development of its institutional and individual capacity to sustain the JEDI effort. Finally, a finding of Transforming meant that the ICRE has fully institutionalized JEDI into the fabric of its being, and would continue to assess its efforts to encourage progress and sustainability. Once at the Transforming stage, the ICRE has reached its goals for institutionalizing JEDI into the fabric of the institute, but with the recognition that new goals should then be set to ensure a dynamic and evolving commitment to progress in JEDI. To illustrate, the developmental progression for one component looked like this: Emerging: There are no ICRE-wide definitions for justice, equity, diversity, and inclusion. Developing: There are definitions for justice, equity, diversity, and inclusion at the ICRE, but there is some variance and inconsistency in their application. Transforming: The ICRE has formal, universally accepted definitions for justice, equity, diversity, and inclusion that are applied consistently across many or most aspects of the ICRE, and which are integral to the conception and execution of any new programs or initiatives. (Appendix. ICRE-JEDI Rubric.)

### Recommending Focus Areas

After applying the rubric and determining the ICRE’s level of JEDI institutionalization, each committee collaboratively chose two components from their dimension as focus areas for priority action (one committee, Administrative Leadership, was asked to choose three focus areas, because their dimension had a larger number of components than the others.) Their selections produced a total of 13 possible focus areas. To determine which areas would take priority, the full task force and the task force chair participated in an exercise commonly known as an Impact/Difficulty matrix led by an experienced facilitator. This exercise first asks the group to rank each possible action item according to its potential to positively affect JEDI (impact) relative to each other item in the matrix. Then it asks the group to rank the same items according to the challenge or complexity – in terms of staffing, time, coordination, buy-in – of implementing it (difficulty.) With Impact charted on the *x*-axis of a matrix and Difficulty on the *y*-axis, the group was then able to determine which items were high impact/high difficulty (strategic), high impact/low difficulty (high ROI), low impact/low difficulty (low-hanging fruit), and low impact/high difficulty (lowest ROI). Similar to the task force kickoff meetings, we scheduled two, 1-hour impact/difficulty exercises to enable busy task force members to attend at least one. After the two exercises were complete, the task force chair combined the results into one table.

## Results

By the end of August 2022, the task force (Table 1.) had completed its charge. The rubric had been customized to the ICRE context and used to measure the ICRE’s current level of institutionalization of JEDI. (Appendix. ICRE-JEDI Rubric.) Each committee had recommended priority components (Table 2.), and the task force had organized these items as Lowest ROI, Strategic, Low-Hanging Fruit, or Highest ROI via the Importance/Difficulty exercise. (Table 3.) On October 20, 2022, representatives of the task force met with ICRE Director Rubio. They presented their report and recommended that she choose two or three specific action items and create a timeline to achieve them. During the meeting, Director Rubio chose four items, two of which were from the “low-hanging fruit” category and could be implemented fairly quickly, one from the “highest ROI” category, and the fourth from the “strategic” category to be completed within one year. (Table 4.) Dr Rubio also accepted the task force’s recommendation that ICRE leadership (1) choose two or three focal components from the matrix each fiscal year, (2) create a plan and timeline to achieve them, (3) publish these plans on the ICRE web site to provide transparency and to allow the community at large to hold the ICRE accountable for progress [27], and (4) reapply the rubric in 18 months (April 2024) to evaluate continuing progress.

**Table 1.** The ICRE-JEDI task force

Task force dimension	Committee members	ICRE role(s)
Philosophy and Mission	Michael J. Fine, MD, MSc Colleen A. Mayowski, EdD, MLIS* Thomas R. Radomski, MD, MS	Faculty Faculty Alumni, Faculty
Faculty Support	Chung-Chou H. (Joyce) Chang, PhD Colleen A. Mayowski, EdD, MLIS* Kenneth J. Smith, MD, MS	Faculty Faculty Alumni, Faculty
Curriculum	Kaleab Z. Abebe, PhD Marie K. Norman, PhD Colleen A. Mayowski, EdD, MLIS*	Faculty Faculty Faculty
Staff Engagement	Colleen A. Mayowski, EdD, MLIS* Megan E. Miller, MEd Chelsea N. Proulx, MPH	Faculty Staff Staff
Student Support	Megan Dooley Sarah Merriam, MD, MS Colleen A. Mayowski, EdD, MLIS*	Staff Alumni, Faculty Faculty
Administrative and Leadership Support	Megan E. Miller, MEd Colleen A. Mayowski, EdD, MLIS* Carla L. Spagnoletti, MD, MS	Staff, Administration Faculty Alumni, Faculty

* Denotes task force chair, who participated in each committee.

**Table 2.** Six rubric dimensions and the components within each dimension as identified, assessed, and prioritized by the designated committee.*

Dimension	Components	Current assessment	Priority component?
Philosophy and Mission	Definition of JEDI	Emerging	Y
	Alignment with Mission	Transforming	N
	Strategic Planning	Emerging	Y
	Historical Context	Developing	N
Faculty Support	Knowledge and Awareness	Developing	N
	Involvement and Support	Emerging	Y
	Faculty Leadership	Developing	Y
	Faculty Rewards	Emerging	N
	Faculty Development and Incentives	Emerging	N
Curriculum	Course Level Attention	Emerging	Y
	Track Level Attention	Emerging	N
	Course Audit	Emerging	Y
	Teaching and Learning Resources	Developing	N
	Service	Emerging	N
Staff Support	Knowledge and Awareness	Developing	Y
	Engagement and Involvement	Emerging	Y
	Staff Incentives	Developing	N
	Staff Recognition	Developing	N
Student Support	Knowledge of JEDI	Developing	N
	Awareness of Opportunities to Learn	Emerging	Y
	ICRE Definition of Student Success	Emerging	Y
	Student Engagement	Emerging	N
	Student Incentives and Rewards	Developing	N
Administrative Leadership	Coordination of ICRE Efforts	Transforming	N
	Policy-Making Entities	Developing	N
	Design of Physical Spaces	Emerging	N
	Diversity-Focused Positions	Developing	N
	Hiring and Retention	Developing	N
	Professional Development	Transforming	N
	Funding	Developing	N
	Senior Administrative Leadership	Developing	Y
	Evaluation and Assessment	Developing	Y
	Resource Management	Developing	N
	Specialized Initiatives	Developing	N
	Alumni Affairs	Emerging	Y

* The full ICRE-JEDI Rubric, where each of these components is fully described and operationalized, can be found in the Appendix.

**Table 3.** Results of the impact/difficulty matrix exercise

Lowest ROI (low impact, high difficulty)	Strategic (high impact, high difficulty)
Encourage faculty to integrate JEDI into their course objectives, lecture content, assignments, activities, discussions, etc. Encourage staff to support and advocate for JEDI in their work Identify ways to actively engage with alumni from diverse backgrounds	Create an official strategic plan for advancing JEDI Implement an ongoing, systematic effort to assess JEDI efforts Ask faculty to conduct a JEDI audit of their courses and instructional materials Develop a formal definition of student success that includes JEDI
Low-Hanging Fruit (low impact, low difficulty)	Highest ROI (high impact, low difficulty)
Promote JEDI workshops and other training opportunities to students Expect ICRE faculty leaders to model JEDI involvement Encourage faculty to support and advocate for JEDI in their work Encourage staff to attend JEDI training and provide paid time off to do so	Develop ICRE-wide definitions for justice, equity, diversity, and inclusion Ensure ICRE administrative leadership operationalizes its commitment to JEDI

**Table 4.** Impact/difficulty matrix exercise items selected for action

Matrix item	Implementation approach	Timeline
Disseminate opportunities to learn about JEDI workshops and other training opportunities to our students	Dissemination through the ICRE Student e-newsletter	December 8, 2022, thereafter the second Thursday of every month
Encourage all staff to extend JEDI training to include evidence of intersectionality and provide paid time off to do so	Dissemination training opportunities via email as they are identified.	Starting October 21, 2022, and continuing indefinitely
Develop ICRE-wide definitions for justice, equity, diversity, and inclusion	Linked to established University definitions on ICRE website	October 21, 2022
Create an official strategic plan for advancing JEDI in the ICRE	Write strategic plan; provide to ICRE Diversity Committee for approval.	January 2023 and will be complete in October 2023

## Discussion

We believe it is essential for institutions to view justice, equity, diversity, and inclusion as central to their mission and to their standards of excellence, knitting JEDI into the fabric of everything they do, and consistently assessing their efforts to ensure forward progress [21]. For JEDI efforts to be meaningful and sustainable, it is not enough for institutions to add a training here or a program there or even to hire someone to oversee diversity efforts. Indeed, doing so is a recipe for ensuring JEDI initiatives remain marginalized rather than institutionalized. Moreover, because JEDI is a continual work in progress and not a “once-and-done” enterprise, goals should be continually adjusted to reflect evolving social awareness and new opportunities [28].

Our ICRE-JEDI rubric is a concrete step in that direction. Like the DEIA Learning System Framework developed by the CTSA DEIA Task Force [29], a rubric-based effort to institutionalize and assess JEDI can be undertaken regardless of whether a department, division, or institute is at an earlier or later stage of JEDI consciousness. By following the process outlined in this manuscript, the ICRE-JEDI rubric can be customized by any academic entity to meet their unique characteristics and needs.

We acknowledge the limitations of our work to date, however. For example, the ICRE-JEDI rubric does not expressly address representation of persons with disabilities. Moreover, while our task force included several alumni of ICRE programs, it failed to recruit a current ICRE student. We will address these shortcomings in the next iteration. Additionally, members of the task force struggled in some cases to get the wording just right as well as to operationalize all the components. We made a conscious decision not to let “the perfect be the enemy of the good,” moving rapidly, if thoughtfully, rather than allowing extensive wordsmithing and revision. We did so on the logic that the ICRE-JEDI rubric is not intended to be a final product but rather a living document that will change in response to a rapidly changing world. At the time of submission, the ICRE-JEDI rubric had not been tested extensively for reliability or validity beyond face validity; likewise, the NERCHE rubric from which it was adapted does not claim validity or reliability. It will undergo further revision by the ICRE as our goals shift, and be altered by other institutions to address their own unique JEDI needs. Future research plans include assessing how our ICRE community perceives the changes that accompany implementation of the rubric, perhaps through surveys or focus groups.

## Conclusion

As Strand *et al*. note, “Tackling a pervasive societal problem such as racism is daunting, but it is time to act [30].” We believe the rubric and process described here offer a scalable and adaptable model for academic health centers and CTSA hubs to follow as they, like us, seek to institutionalize their values vis-à-vis JEDI, build real buy-in from all their members, assess themselves systematically, and hold themselves accountable for action.

## Supporting information

Norman et al. supplementary material Norman et al. supplementary material

## Supplementary material

The supplementary material for this article can be found at https://doi.org/10.1017/cts.2023.599.
