# Supplementary material for: Time to act: A rubric-based approach for institutionalizing justice, equity, diversity, and inclusion
Source: J Clin Transl Sci. 2023 Jul 24;7(1):e172. doi: 10.1017/cts.2023.599 (PMC10514682; doi:10.1017/cts.2023.599)
Supplement: Norman et al. supplementary material [file S205986612300599Xsup001.pdf]

Self-Assessment Rubric for the Institutionalization of Justice, Equity, Diversity, and Inclusion at the ICRE

Institute for Clinical Research Education

University of Pittsburgh School of Medicine

www.icre.pitt.edu

Contact: Colleen Mayowski

mayowski@pitt.edu

Copyright 2023, Institute for Clinical Research Education, University of Pittsburgh

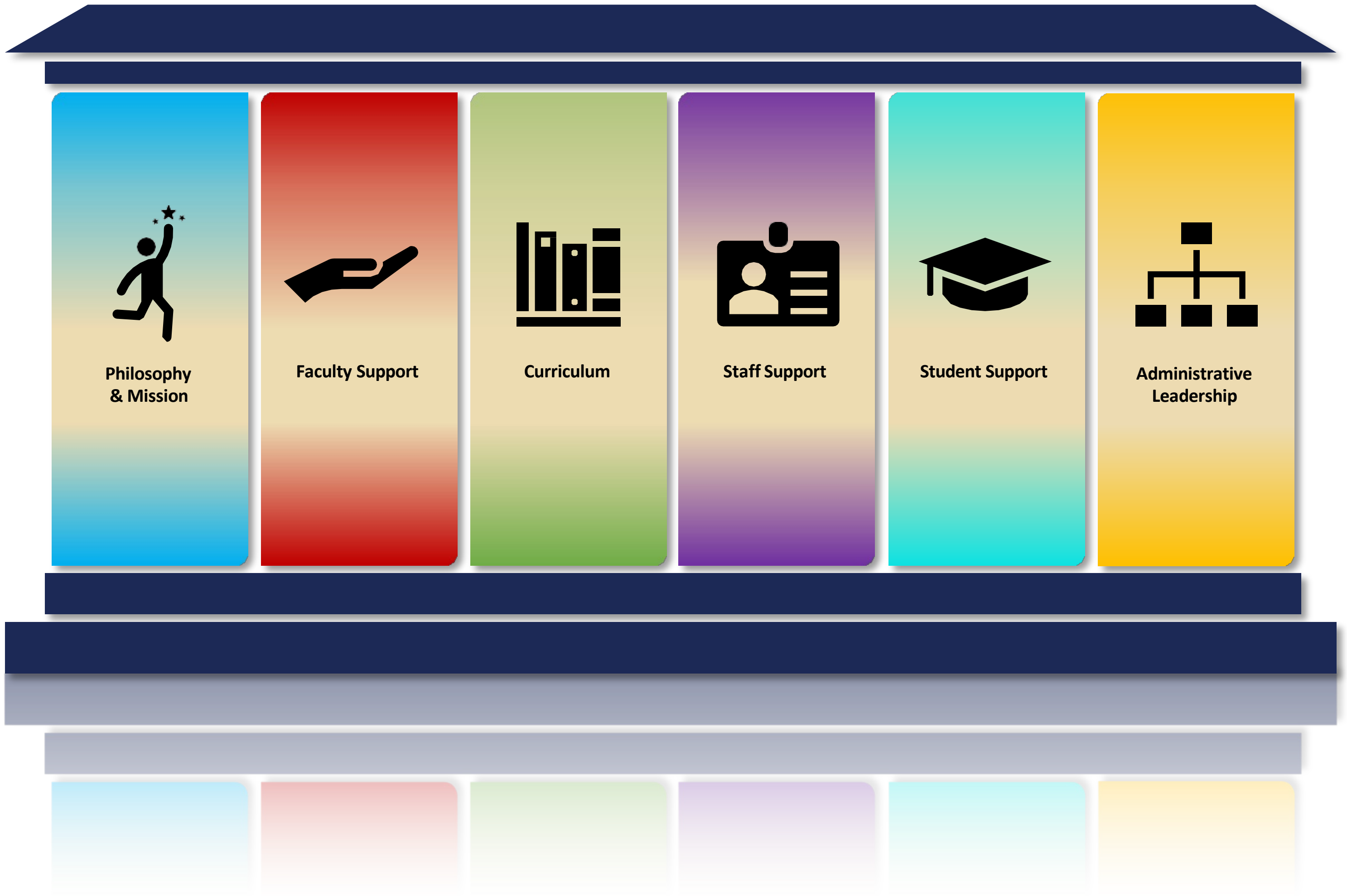

Stages of Development

Stage One: Emerging

At this stage, the ICRE either has insufficient information to draw a conclusion or is just beginning to recognize justice, equity, diversity, and inclusion as strategic priorities and is gathering resources and building constituencies for this effort.

Stage Two: Developing

At this stage, the ICRE is focused on ensuring the development of its institutional and individual capacity to sustain the justice, equity, diversity, and inclusion effort.

Stage Three: Transforming

At this stage, the ICRE has fully institutionalized justice, equity, diversity, and inclusion into the fabric of its being, and continues to assess its efforts to encourage progress and sustainability.

***Once at the transforming stage**, the ICRE has reached its goals for institutionalizing justice, equity, diversity, and inclusion into the fabric of the institute, but it must recognize the ever-changing environment and continue to assess its progress and the sustainability of its achievements as it moves into the future.*

I. PHILOSOPHY AND MISSION OF JUSTICE, EQUITY, DIVERSITY, AND INCLUSION

A primary feature of institutionalized diversity effort is the development of a shared definition for diversity and inclusive excellence that provides meaning, focus, and emphasis for renewal and transformation. How narrowly or broadly justice, equity, diversity, and inclusion (JEDI) are defined with determine who participates, who will provide financial resources and other support, and the degree to which diversity will become intrinsic to ICRE culture.

COMPONENTS	CURRENT STAGE	STAGE ONE: Emerging	STAGE TWO: Developing	STAGE THREE: Transforming
**DEFINITION OF JUSTICE, EQUITY, DIVERSITY, AND INCLUSION**	**Emerging (Focus Point)**	There are no ICRE-wide definitions for justice, equity, diversity, and inclusion.	There are definitions for justice, equity, diversity, and inclusion at the ICRE, but there is some variance and inconsistency in their application.	The ICRE has formal, universally accepted definitions for justice, equity, diversity, and inclusion that are applied consistently across many or most aspects of the ICRE, and which are integral to the conception and execution of any new programs or initiatives.
**ALIGNMENT WITH ICRE MISSION**	**Transforming**	While justice, equity, diversity, and inclusion complement many aspects of the ICRE's mission, they remain on the periphery. Justice, equity, diversity, and inclusion are rarely included in larger efforts that focus on the core mission of the ICRE. An official mission statement does not exist.	Justice, equity, diversity, and inclusion are often mentioned as a primary or important part of the ICRE's mission, but are not included in the official mission statement.	Justice, equity, diversity, and inclusion are fully integrated into the mission statement of the ICRE, and the mission statement is publicly disseminated.
**STRATEGIC PLANNING**	**Emerging (Focus Point)**	The ICRE does not have an official strategic plan for advancing justice, equity, diversity, and inclusion at the ICRE.	Although certain short-range and long-range goals for justice, equity, diversity, and inclusion have been defined for the ICRE, these goals have not been formalized into an official strategic plan that will guide the implementation of these goals.	The ICRE has developed an official, publicly available strategic plan for advancing justice, equity, diversity, and inclusion at the ICRE, which includes viable short-range and long-range institutionalization goals, using process-oriented and outcomes-oriented quality indicators and metrics.
**HISTORICAL CONTEXT**	**Developing**	Justice, equity, diversity, and inclusion and their complex relationship to health disparities, white supremacy in research, systemic racism, and the geographic or cultural history of the community are not acknowledged nor widely understood.	Justice, equity, diversity, and inclusion and their complex relationship to health disparities, white supremacy in research, systemic racism, and the geographic or cultural history of the community are inconsistently acknowledged, understood, or integrated into appropriate curricular content.	Justice, equity, diversity, and inclusion and their complex relationship to health disparities, white supremacy in research, systemic racism, and the geographic or cultural history of the community are fully acknowledged, widely understood, integrated into appropriate curricular content, and used to build an inclusive present and future.

II. FACULTY SUPPORT FOR AND INVOLVEMENT IN JUSTICE, EQUITY, DIVERSITY, AND INCLUSION

An important element for justice, equity, diversity, and inclusion institutionalization is the degree to which the faculty take ownership of justice, equity, diversity, and inclusion (JEDI) as essential to the academic core of the ICRE.

COMPONENTS	CURRENT STAGE	STAGE ONE: Emerging	STAGE TWO: Developing	STAGE THREE: Transforming
**FACULTY KNOWLEDGE AND AWARENESS**	**Developing**	Less than 60% of faculty members have completed formalized, evidence-based JEDI training or workshops sponsored by a credible organization or source.	60-99% of faculty members have completed formalized, evidence-based JEDI training or workshops sponsored by a credible organization or source.	All faculty members have completed formalized, evidence-based JEDI training or workshops sponsored by a credible organization or source. Evidence of intersectional training or multiple trainings that show evidence of different perspectives must be present.
**FACULTY INVOLVEMENT & SUPPORT**	**Emerging (Focus Point)**	Less than 60% of faculty members are instructors, supporters, or advocates of justice, equity, diversity, and inclusion.<br><br>Few (<60%) support the strong infusion of justice, equity, diversity, and inclusion into the academy or into their own professional work. A few faculty (<60%) members at the ICRE sustain justice, equity, diversity, and inclusion activities.<br><br>Faculty have not examined their syllabi for opportunities to incorporate JEDI.	While 60-99% of faculty members are instructors, supporters, or advocates of justice, equity, diversity, and inclusion, few (<60%) are advocates for infusing diversity in the overall mission and/or their own professional work. An inadequate or unsatisfactory number (<60%) of faculty members are engaged in justice, equity, diversity, and inclusion activities. Few faculty have examined their syllabi for opportunities to incorporate JEDI.	100% of faculty members participate as instructors, supporters, and advocates of justice, equity, diversity, and inclusion and support the infusion of justice, equity, diversity, and inclusion both into the ICRE's overall mission and the faculty members' individual professional work. All have examined their syllabi for opportunities to incorporate JEDI.
**FACULTY LEADERSHIP**	**Developing (Focus Point)**	Less than 60% of ICRE faculty leaders (for example, track directors, course directors, program directors, etc.) encourage the advancement of justice, equity, diversity, and inclusion at the ICRE and are not encouraging colleagues to engage in diversity work at the ICRE or in their courses.	60-99% of ICRE faculty leaders (for example, track directors, course directors, program directors, etc.) encourage the advancement of the ICRE's justice, equity, diversity, and inclusion efforts and encourage colleagues to engage in justice, equity, diversity, and inclusion work at the ICRE or in their courses.	100% of ICRE faculty leaders (for example, track directors, course directors, program directors, etc.) encourage the advancement of the ICRE's justice, equity, diversity, and inclusion efforts and/or advocate and encourage colleagues to engage in justice, equity, diversity, and inclusion work at the ICRE or in their courses.
**FACULTY REWARDS**	**Emerging**	In general, faculty members simply document activities in justice, equity, diversity, and inclusion service work; faculty members' work in JEDI is not recognized during the annual review process, and in recommendation for tenure, or promotion and sabbatical processes by DGIM or ICRE.	Although faculty members are encouraged to go beyond simply documenting JEDI activities and are encouraged to pursue justice, equity, diversity, and inclusion activities in their work, this is not always recognized during the ICRE or DGIM's review, tenure, promotion, or sabbatical processes.	Faculty are encouraged to pursue diversity, equity, inclusion and justice activities. Faculty who are involved in justice, equity, diversity, and inclusion receive recognition for it during the ICRE or DGIM's review, tenure, promotion, or sabbatical processes.
**FACULTY DEVELOPMENT AND INCENTIVES**	**Emerging**	There are minimal opportunities and dedicated funds to support and sustain faculty capacity specifically for JEDI-related work over time. There are minimal incentives provided (e.g., mini-grants, course releases, funds for conferences, etc.) to pursue JEDI activities.	There are some opportunities and dedicated funds to support and sustain faculty capacity specifically to do JEDI-related work over time. There are some incentives provided (e.g., mini-grants, course releases, funds for conferences, etc.) to pursue JEDI activities.	There are many opportunities and dedicated funds to specifically support and sustain faculty capacity to do justice, equity, diversity, and inclusion-related work over time. There are many incentives provided (e.g., mini-grants, course releases, funds for conferences, etc.) to pursue JEDI activities.

III. CURRICULUM: TEACHING AND SERVICE SUPPORTING JUSTICE, EQUITY, DIVERSITY, AND INCLUSION

One of the essential factors for institutionalizing justice, equity, diversity, and inclusion at the ICRE is the degree to which faculty are involved in the implementation and advancement of epistemologies, pedagogies, and service related to justice, equity, diversity, and inclusion (JEDI).

COMPONENTS	CURRENT STAGE	STAGE ONE: Emerging	STAGE TWO: Developing	STAGE THREE: Transforming
**COURSE LEVEL ATTENTION TO JEDI**	**Emerging (Focus Point)**	There is deliberate attention to JEDI in the course objectives, content, assignments, activities, and classroom ground rules for discussions in 49% or fewer of the courses offered at the ICRE.	There is deliberate attention to JEDI in the course objectives, content, assignments, activities, and classroom ground rules for discussions in 50% or more but not all of the courses offered at the ICRE.	There is deliberate attention to JEDI in the course objectives, content, assignments, activities, and classroom ground rules for discussions in every course offered at the ICRE.
**TRACK LEVEL ATTENTION TO JEDI**	**Emerging**	The track director does not have a vision or strategy for incorporating JEDI into the track and cannot articulate the relevance of JEDI to the track	The track director demonstrates some vision or strategy for incorporating JEDI into the track and can articulate some the relevance of JEDI to the track	The track director demonstrates a strong vision and strategy for incorporating JEDI into the track and can make a strong case for why JEDI is relevant to the goals of the track
**COURSE AUDIT**	**Emerging (Focus Point)**	Fewer than 50% of faculty members have performed a course audit designed to evaluate inclusivity of their instructional materials.	At least 50% but not all faculty members have performed a course audit designed to evaluate inclusivity of their instructional materials.	All faculty members have performed a course audit designed to evaluate inclusivity of their instructional materials.
**TEACHING AND LEARNING RESOURCES**	**Developing**	The ICRE rarely (1-2x per year) if ever promotes resources and programs designed to help faculty respond effectively to the diverse experiences of all students in an ICRE course.	The ICRE sometimes (3-5x per year) promotes resources and programs designed to help faculty respond effectively to the diverse experiences of all students in an ICRE course.	The ICRE sometimes (>5x per year) promotes resources and programs designed to help faculty respond effectively to the diverse experiences of all students in an ICRE course.
**SERVICE**	**Emerging**	Fewer than 50% of faculty perform some service to the department or institution that incorporates diversity, equity, inclusion, and/or justice (diversity committee, task forces, other initiatives).	At least 50% but not all faculty perform some service to the department or institution that incorporates diversity, equity, inclusion, and/or justice (diversity committee, task forces, other initiatives).	All faculty perform some service to the department or institution that incorporates diversity, equity, inclusion, and/or justice (diversity committee, task forces, other initiatives).

IV. STAFF ENGAGEMENT AND INVOLVEMENT IN JUSTICE, EQUITY, DIVERSITY, AND INCLUSION

One of the essential factors for institutionalizing diversity in higher education is the degree to which staff members are involved in implementation and advancement of justice, equity, diversity, and inclusion (JEDI) issues at the ICRE.

COMPONENTS	CURRENT STAGE	STAGE ONE: Emerging	STAGE TWO: Developing	STAGE THREE: Transforming
**STAFF KNOWLEDGE AND AWARENESS**	**Developing (Focus Point)**	Less than 60% of staff members have completed formalized, evidence-based JEDI training or workshops sponsored by a credible organization or source.	60-99% of staff members have completed formalized, evidence-based JEDI training or workshops sponsored by a credible organization or source.	All staff members have completed formalized, evidence-based JEDI training or workshops sponsored by a credible organization or source. Evidence of intersectional training or multiple trainings that show evidence of different perspectives must be present.
**STAFF ENGAGEMENT AND INVOLVEMENT**	**Emerging (Focus Point)**	Less than 60% of staff members are recognized supporters, instructors, or advocates of justice, equity, diversity, and inclusion. A few staff members at the ICRE sustain these activities.	60-99% of staff members are recognized supporters, instructors, or advocates of justice, equity, diversity, and inclusion, and sustain these activities.	All staff members participate as supporters, instructors and advocates of justice, equity, diversity, and inclusion and many infuse these both into the ICRE's overall mission and the staff members' individual professional work.
**STAFF INCENTIVES**	**Developing**	In general, staff members are not encouraged to engage in justice, equity, diversity, and inclusion work; few if any incentives are provided (e.g., funds for conferences, work release for professional development etc.) to pursue related activities.	Staff members are reminded at the time of their self-appraisal of opportunities to participate in JEDI activities and are provided various incentives (funds for justice, equity, diversity, and inclusion conferences, work release for professional development, etc.) to pursue related activities.	Staff members are actively, regularly reminded and encouraged to engage in JEDI work and are provided various incentives (funds for related conferences, work release for professional development, etc.) to pursue justice, equity, diversity, and inclusion activities.
**STAFF RECOGNITION**	**Developing**	Staff members' work in justice, equity, diversity, and inclusion is not recognized during their performance review and promotion process or in a public capacity or sphere (newsletter, Twitter, etc.).	Staff members' work in justice, equity, diversity, and inclusion is recognized during their annual performance review and promotion process, and there may be recognition in a public capacity or sphere.	Staff members who engage in justice, equity, diversity, and inclusion practices receive ongoing recognition from ICRE leadership and in a public capacity or sphere (newsletter, Twitter, etc.).

V: STUDENT SUPPORT FOR AND INVOLVEMENT IN JUSTICE, EQUITY, DIVERSITY, AND INCLUSION

An important element of institutionalization is the degree to which students are provided with opportunities to learn about justice, equity, diversity, and inclusion (JEDI) in co-curricular settings; are aware of these opportunities; engage in these opportunities; and play a leadership role in the development of JEDI at the ICRE.

COMPONENTS	CURRENT STAGE	STAGE ONE: Emerging	STAGE TWO: Developing	STAGE THREE: Transforming
**STUDENT KNOWLEDGE OF JEDI**	**Developing**	Less than 60% of students can articulate what justice, equity, diversity, and inclusion are nor understand why knowledge and experience in these areas are essential to their education and their future work.	60 to 80% of students can articulate what justice, equity, diversity, and inclusion are and understand why knowledge and experience in these areas are essential to their education and their future work.	81 to 100% of students know what justice, equity, diversity, and inclusion are. They understand and can articulate why knowledge and experience in these areas are essential to their education and their future work.
**STUDENT AWARENESS OF OPPORTUNITIES TO LEARN**	**Emerging (Focus Point)**	Less than 60% of ICRE students can identify an opportunity to participate in co-curricular JEDI training, activities, or workshops sponsored by a credible organization or source.	60 to 80% of ICRE students can identify an opportunity to participate in co-curricular JEDI training, activities, or workshops sponsored by a credible organization or source.	81 to 100% of students can name multiple opportunities to participate in co-curricular JEDI training, activities, or workshops sponsored by a credible organization or source.
**ICRE DEFINITION OF STUDENT SUCCESS**	**Emerging (Focus Point)**	The ICRE does not have a definition of student success that supports linkages between justice, equity, diversity, and inclusion and student success.	A definition of student success that supports linkages between justice, equity, diversity, and inclusion and student success exists, but it is not widely known or disseminated.	The ICRE has an articulated definition of student success that supports linkages between justice, equity, diversity, and inclusion and student success. It is widely understood and publicly disseminated.
**STUDENT ENGAGEMENT**	**Emerging**	Less than 60% of students are involved or engaged in JEDI activities, and engage rarely (once a year).	60 to 80% of ICRE students are involved or engaged in JEDI activities, and engage often (every semester).	81 to 100% of students are involved or engaged in JEDI activities, and engage often (every semester). Some may even participate in a leadership capacity.
**STUDENT INCENTIVES AND REWARDS**	**Developing**	The ICRE has neither formal mechanisms (e.g. notation on students' transcripts, etc.) nor informal mechanisms (news stories in paper, unofficial student certificates of achievement) that encourage students	The ICRE offers only informal mechanisms (news stories in paper, unofficial student certificates of achievement) that encourage students to participate in justice, equity, diversity, and inclusion activities or research, or only formal mechanisms (e.g. notation on students' transcripts, etc.)	The ICRE has both formal and informal mechanisms in place that encourage students to participate in justice, equity, diversity, and inclusion activities or research and provides recognition for their participation in these efforts throughout the ICRE.

VI: ADMINISTRATIVE AND LEADERSHIP SUPPORT FOR JUSTICE, EQUITY, DIVERSITY, AND INCLUSION

In order for justice, equity, diversity, and inclusion (JEDI) to become institutionalized at the ICRE, senior leadership must demonstrate commitment and ensure that the ICRE provides substantial resources, support, and accountability toward the effort.

COMPONENTS	CURRENT STAGE	STAGE ONE: Emerging	STAGE TWO: Developing	STAGE THREE: Transforming
**COORDINATION OF ICRE EFFORTS**	**Transforming**	There is no formal ICRE entity (e.g., community of practice, committee, center, office, or clearinghouse) charged with coordinating the implementation, advancement, and institutionalization of justice, equity, diversity, and inclusion.	There is a formal ICRE entity charged with coordinating various constituencies in the implementation, advancement, and institutionalization of justice, equity, diversity, and inclusion (e.g., community of practice, committee, center, office, or clearinghouse), but the entity provides services only to a certain constituency (e.g., students, faculty) or limited part of the ICRE (e.g., certain degrees or tracks).	The ICRE supports a coordinating entity actively engaged in assisting ICRE constituencies in the design, implementation, advancement, and institutionalization of justice, equity, diversity, and inclusion (e.g., community of practice, committee, center, office, or clearinghouse).
**POLICY-MAKING ENTITIES**	**Developing**	The ICRE's official and influential policy-making entities/ board(s)/committee(s) do not recognize justice, equity, diversity, and inclusion as essential educational goals for the ICRE.	The ICRE's official and influential policy-making entities/board(s)/committee(s) recognize justice, equity, diversity, and inclusion as essential educational strategies, but no formal policies have been developed.	The ICRE's policy-making entities, board(s)/committee(s) recognize justice, equity, diversity, and inclusion as essential educational strategies and formal policies have been developed or implemented.
**DESIGN OF PHYSICAL SPACES TO FOSTER INCLUSION**	**Emerging**	Physical workspaces are designed and constructed without regard to research and best practices for addressing issues of JEDI.	Research and best practices for addressing issues of JEDI are considered when designing and constructing physical workspaces, but are not consistently implemented.	The ICRE researches and consistently implements best practices for designing and constructing physical workspaces shown to be highly effective at bringing and keeping individuals who add to its diverse workforce.
**DIVERSITY-FOCUSED POSITIONS**	**Developing**	There is not a senior administrator/faculty member with formal responsibility for advancing justice, equity, diversity, and inclusion across the ICRE.	There are positions with formal responsibility for advancing justice, equity, diversity, and inclusion exist; however, decision-making authority for these individuals may be limited and not consistently integrated into senior-level decision-making.	There is a senior administrator or faculty member who has formal responsibility for advancing justice, equity, diversity, and inclusion across the ICRE. This individual has senior-level decision-making authority equal to other administrative peers within the leadership team; the individual is integral to all ICRE renewal and transformation efforts.
**HIRING & RETENTION**	**Developing**	Hiring and retention policies and procedures do not address justice, equity, diversity, and inclusion considerations.	Policies and procedures are designed to hire and train a diverse workforce but have proven only partially successful. Policies are not documented in writing.	The ICRE has developed hiring and retention policies and procedures that have proven to be highly effective at bringing and keeping individuals who add to its diverse workforce, and has codified these in writing.
**PROFESSIONAL DEVELOPMENT**	**Transforming**	Professional development sponsored by a credible organization or source and designed to prepare faculty and staff to adequately meet the needs of a diverse, equitable, inclusive, and just ICRE is lacking.	Professional development sponsored by a credible organization or source and designed to prepare faculty and staff to meet the needs of a diverse, equitable, inclusive, and just ICRE is available, but it is either inadequate or ineffective.	Faculty and staff have access to an adequate array of effective professional development programs sponsored by a credible organization or source, to prepare them to meet the needs of a diverse, equitable, inclusive, and just ICRE.

VI: ADMINISTRATIVE AND LEADERSHIP SUPPORT FOR JUSTICE, EQUITY, DIVERSITY, AND INCLUSION (Continued)

In order for justice, equity, diversity, and inclusion (JEDI) to become institutionalized at the ICRE, senior leadership must demonstrate commitment and ensure that the ICRE provides substantial resources, support, and accountability toward the effort.

COMPONENTS	CURRENT STAGE	STAGE ONE: Emerging	STAGE TWO: Developing	STAGE THREE: Transforming
**FUNDING**	**Developing**	The ICRE's justice, equity, diversity, and inclusion activities are supported primarily by soft money (short-term grants) from sources outside the ICRE.	The ICRE's justice, equity, diversity, and inclusion activities are supported by both soft money (short-term grants) from sources outside the ICRE as well as hard money from the ICRE.	Primarily operational (hard money) or endowed funding from the ICRE supports the ICRE's justice, equity, diversity, and inclusion activities.
**SENIOR ADMINISTRATIVE LEADERSHIP**	**Developing (Focus Point)**	The ICRE's administrative leaders have a limited and/or contradictory understanding of justice, equity, diversity, and inclusion, and are unaware that it is an essential component of education, often confusing it with affirmative action.	Administrative leaders share an expansive understanding of justice, equity, diversity, and inclusion and its importance, but do not yet have a consistent plan to operationalize it.	Administrative leadership functions as a unified team which consistently operationalizes its commitment to justice, equity, diversity, and inclusion as an essential feature in its renewal efforts.
**ICRE EVALUATION & ASSESSMENT**	**Developing (Focus Point)**	There is no organized ICRE effort underway to account for the number and quality of justice, equity, diversity, and inclusion activities taking place (e.g. climate assessments, student diversity learning outcomes, standardized measures).	An action plan for assessment of justice, equity, diversity, and inclusion initiatives across the ICRE has been proposed but research of such data has not been initiated nor disseminated.	An ongoing, systematic effort is in place to implement, evaluate, and recalibrate the assessment of diversity efforts that are taking place throughout the ICRE. Research has been initiated and is currently being disseminated throughout the ICRE.
**ICRE RESOURCE MANAGEMENT**	**Developing**	Little concrete evidence exists that ICRE policy and decision-making in resource management (including budgeting, purchasing, vendor management, and human resource planning) integrates an ICRE-wide value for justice, equity, diversity, and inclusion, while complying with University policies and procedures.	Some evidence exists that policy and decision-making in resource management (including budgeting, purchasing, vendor management, and human resource planning) integrates an ICRE-wide value for justice, equity, diversity, and inclusion, while complying with University policies and procedures.	Policy and decision-making in resource management (including budgeting, purchasing, vendor management, and human resource planning) is characterized by a consistent, ICRE-wide value for justice, equity, diversity, and inclusion, while complying with University policies and procedures.
**SPECIALIZED ICRE INITIATIVES**	**Developing**	Specialized goal-oriented initiatives (career development programs, grants, degree and certificate programs) do not take into account ICRE justice, equity, diversity, and inclusion efforts.	Specialized initiatives (career development programs, grants, degree and certificate programs) sometimes integrate justice, equity, diversity, and inclusion efforts occurring at the ICRE but with no reliable consistency.	Specialized initiatives—regardless of the topic addressed—always support and integrate the established commitment to justice, equity, diversity, and inclusion across the ICRE.
**ALUMNI AFFAIRS**	**Emerging (Focus Point)**	The ICRE seldom or never engages with alumni who represent diverse populations within the larger community, except for data collection purposes.	The ICRE sometimes engages with alumni (for example, with an ICRE newsletter of at least annual frequency) who represent diverse populations within the larger community.	The ICRE has an active, reciprocal relationship with alumni who represent diverse populations within the community. This engaged work, for example, may include an active donor base, board representation, and/or involvement in curricular and co-curricular activities as consultants, advisors, and participants.
